# Associations of Exhaled Carbon Monoxide and Fractional Exhaled Nitric Oxide with Metabolic Syndrome: A Cohort Study

**DOI:** 10.1038/srep24532

**Published:** 2016-04-14

**Authors:** Yanjun Guo, Jixuan Ma, Wei Lu, Jintong He, Runbo Zhang, Jing Yuan, Weihong Chen

**Affiliations:** 1 Department of Occupational and Environmental Health, School of Public Health, Tongji Medical College, Huazhong University of Science and Technology, Wuhan, China; 2 Key Laboratory of Environment and Health in Ministry of Education & Ministry of Environmental Protection, State Key Laboratory of Environmental Health (Incubating), School of Public Health, Tongji Medical College, Huazhong University of Science and Technology, Wuhan, China

## Abstract

Exhaled carbon monoxide (eCO) and fractional exhaled nitric oxide (FeNO) could reflect underlying inflammatory and oxidative stresses, which play important roles in pathogenetic pathways of metabolic syndrome (MetS). However, epidemiologic evidence was limited. We conducted a study in Wuhan-Zhuhai (WHZH) cohort of 3649 community participants to investigate the association between eCO, FeNO and MetS in both cross-sectional and prospective ways. The results showed that higher eCO and FeNO were associated cross-sectionally with a higher prevalence of MetS. The multivariable-adjusted odds ratios for MetS at baseline were 1.22 (95% confidence interval [CI]: 1.11 to 1.35) associated with per log eCO and 1.14 (95% CI: 1.00 to 1.30) associated with per log FeNO. During a follow-up of 3 years, 358/2181 new developed MetS cases were identified. Compared with lowest quartile of eCO and FeNO, the multivariable-adjusted risk ratios (95% CI) for MetS were 1.48 (1.06 to 2.06) related to the highest quartile of eCO. These findings remained consistent across sex but not smoking status, eCO was only associated with MetS in non-smokers when stratified by smoking status. In conclusion, our study demonstrated that eCO and FeNO were independently and positively associated with the prevalence of MetS cross-sectionally, while only eCO was positively related with the incidence of MetS prospectively.

Exhaled carbon monoxide (eCO) and fractional exhaled nitric oxide (FeNO)^1^ are both small gas molecules produced endogenously in human body. The endogenous eCO is the by-product of the degradation of heme through the heme oxygenase (HO) enzyme system in cells and tissues^2^,^3^,^4^. HO catalyzes the first and rate-limiting step in the oxidative degration of heme to ferrous iron, biliverdin-IXα, and CO^5^. Although, the biochemical characterization of endogenous CO production is precise, eCO have been regarded as metabolic waste for a long time till the discovering of physiologically similarity with nitric oxide (NO)^5^,^6^. FeNO is synthesized by one of the three forms of nitric oxcide synthases (NOS), which catalyze the transformation of L-arginine to L-citrulline and NO in the presence of oxygen and several cofactors^7^,^8^,^9^. And it has been recommended as a monitor of airway inflammation in asthma patients by US Food and Drug Administration (FDA)^1^. In addition, NO is a mediators of vasodilation, regulators of endothelial cell proliferation, and maintainers of vascular health^10^,^11^.

Accumulating evidences suggest eCO and FeNO are structurally and biologically similar in the ability to modulate vascular functions and cellular homeostasis^5^,^12^,^13^,^14^, and are evaluated as candidate breath biomarker of pathophysiological states^4^,^13^,^14^,^15^. Both eCO and FeNO can be cytoprotective and exert antioxidant, antiinflammatory, and antiapoptotic properties at normally physiological levels^1^,^8^,^16^, while, excess eCO and FeNO can reflect underlying inflammatory and oxidative stress.

Metabolic syndrome (MetS), characterized by a constellation of multiple cardiometabolic abnormalities including central obesity, hypertension, dyslipidemia, and impaired glucose tolerance, has been recognized as a condition strongly predicting type 2 diabetes and cardiovascular disease (CVD)^17^. MetS shares common pathogenetic pathways with CVD and type 2 diabetes, where oxidative stress and inflammation play central roles^8^,^9^,^18^,^19^. Therefore, exhaled biomarkers like endogenously CO and NO, reflecting increment in oxidative stress, systemic inflammation and endothelial dysfunction can be studied as potential non-invasive tools to predict MetS.

However, there are limited epidemiologic studies to investigate the relationship between eCO, FeNO and MetS up to date. Additionally, the concentrations of eCO and FeNO were indicated to be significant affected by sex and smoking status^13^. But no study has been conducted to examine the effects of these factors on the association of eCO and FeNO with MetS. Therefore, we sought to investigate the association of eCO, FeNO with MetS in both cross-sectionally and longitudinally ways, as well as evaluating whether the association is modified by sex and smoking status in a community-based cohort in China.

## Results

The baseline characteristics of our participants were presented in Table 1 according to quartiles of eCO and FeNO. Rug plots displaying the distribution of eCO and FeNO of our sample were shown in Supplementary Fig. S1 and Supplementary Fig. S2. All the participants aged from 18 to 80 years, and the average age was 52.7. Female participants took up 65.1% of the whole population. During the 3 years of follow-up, we identified 358 new MetS cases out of 2,181participants. We observed increases of waist circumferences, low-density lipoprotein cholesterol (LDL), serum triglycerides and blood glucose across increasing eCO and FeNO quartiles. In this study, the levels of eCO and FeNO were higher in men and smokers. There were also increases in age and BMI across increasing FeNO quartiles.

The cross-sectional associations of eCO and FeNO with MetS were shown in Table 2. After adjusting for multiple potential confounders, a positive relationship was indicated between eCO, FeNO and the prevalence of MetS. Comparing with the lowest quartile, the multi-variate adjusted ORs (95%CI) for MetS were 0.99 (0.80–1.23), 1.03 (0.83–1.28) and 1.71 (1.34–2.18) from the second quartile to the fourth quartile of eCO, and the OR (95%CI) was 1.22 (1.11–1.35) correspond to 1-unit change in log eCO. Compared with the lowest quartile, the ORs (95%CI) for MetS were 1.37 (1.07–1.74), 1.16 (0.91–1.49) and 1.45 (1.13–1.86) across the increasing quartiles of FeNO, and the OR (95%CI) was 1.14 (1.00–1.30) for 1-unit increase in log FeNO.

**Figure 1:**
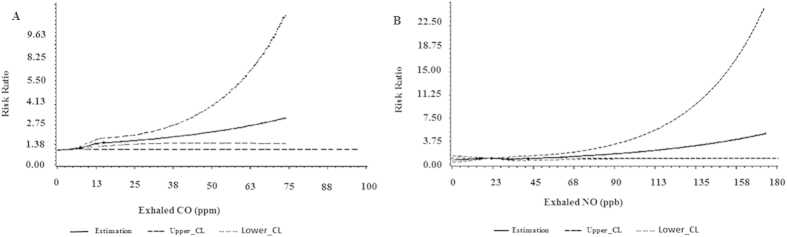
Multivariable-adjusted spline graph displaying the relation of eCO (**A**) and FeNO (**B**) with MetS.

The associations were further investigated longitudinally (Table 3). There was still a positive relationship between eCO and the development of metabolic syndrome. The risk ratio (RR) and 95%CI were 1.23 (0.87–1.74), 1.35 (1.01–1.82) and 1.48 (1.06–2.06) as the increasing of eCO quartiles in multi-variable model. And the RR (95%CI) correspond to 1-unit change in log eCO was 1.12 (1.02–1.26). However, there was no significance in the relationship between FeNO and MetS across the increasing quartiles of FeNO. In the spline regression analysis, the associations were mainly the same (Fig. 1). Additionally, the association between FeNO and MetS remained consistent in stratified analysis according to sex and smoking status, but the association between eCO and MetS was modified by smoking status; eCO was related to MetS only in non-smokers (see Supplementary Table S3).

## Discussion

To our knowledge, this was the first cohort study to investigate the associations of eCO and FeNO with MetS. Our principle findings showed that: higher eCO and FeNO were associated cross-sectionally with a higher prevalence of MetS under adjustment for possible confounders; and, higher eCO was also associated with further development of MetS. In stratified analysis, eCO was related to MetS in non-smokers, but the association was not modified by sex and smoking status for FeNO and MetS.

The eCO and FeNO has evolved rapidly as markers of breath analysis in the past decade. Breath analysis has the potential to offer relatively inexpensive, rapid, noninvasive methods for detecting and monitoring a variety of diseases^8^,^20^. And the ideal requirement of an exhaled marker needs to be easy to perform measurement with a simple methodology accomplishing reproducibility and repeatability of measurement^9^. Therefore, eCO and FeNO have been taken as markers of pulmonary and airway inflammation for a long time^2^,^11^,^12^,^21^,^22^. In recent years, the relationship between eCO, FeNO and metabolic diseases has drawn great attention^9^,^19^,^23^,^24^,^25^.

In this study, eCO was cross-sectionally and prospectively associated with Mets and the association remained consistent across different sex and modified by smoking status subgroups. Similar association was also indicated by Cheng and his collegues in a community-based study, which reported that interindividual variation in CO was associated with metabolic derangements and the development of manifest CVD^13^. Several mechanisms may contribute to the associations. Firstly, the HO-dependent response to oxidative stress and inflammation may be a main common pathway by which CO is associated with metabolic traits. Endogenous CO is a byproduct of heme oxygenase (HO) activity, and oxidative stress and inflammatory cytokines can increase the activity of HO^3^,^26^,^27^. Excess levels of CO could disrupt the mitochondrial electron transport chain, resulting in the generation of reactive oxygen species. On the other hand, studies suggested that both hyperglycemia and the progressive accumulation of advanced glycation end-products in diabetes mellitus can be drivers of increased oxidative activity and, in turn, long-term activation of HO and oxidized lipids also activate HO may be related to the relation of dyslipidemia with excess CO levels^28^. Totally, excess endogenous CO may be both the cause and results of oxidative stress and inflammation. Secondly, CO has also been shown to stimulate insulin and glucagon release from islet cells as well as modulate insulin sensitivity and glucose tolerance in metabolic disorder animals^29^,^30^. In our study, eCO levels were also indicated to be higher in smoker groups which were consistent with former studies^13^,^31^,^32^. Cheng and his collegues also found the differences in their study, but they didn’t investigate whether the association between eCO and MetS differed by gender and smoking status^13^. Although, the association between eCO and MetS was not modified by sex, it was modified by smoking status. eCO was only significantly related with MetS in non-smokers after adjusting for multiple potential confounders. This can be partly explained by the relatively smaller population of smokers compared to non-smokers in this population, for we also observed increased but not significantly risk in smokes. Another mechanism might be that the dependence of endogenous CO production on HO activity, for HO activity can be modulated by smoking and smoking can lead to the changes of microenvironment which also have impact on the expression and inducibility of HO^33^.

To our knowledge, no previous study was conducted to explore the association between FeNO levels with MetS, although endogenous NO is regarded as a regulatory factor that plays a key role in many different physiologic and pathophysiological processes. In our study, FeNO was found to be positively associated with MetS cross-sectionally but not in prospective analysis. As FeNO was also found to be higher in male and smoker groups, which was indicated in previous studies^10^,^21^,^34^,^35^. The association between FeNO and MetS remained consistent when stratified by sex and smoking status in our study. FeNO associated with metabolic disease mainly through the endothelium dysfunction^36^, which has been advocated as a pathogenic mechanism of metabolic diseases^9^,^37^. FeNO can also associate with metabolic disease through asymmetric dimethylarginine (ADMA), a common link between oxidative stress, inflammation and metabolic syndrome, by competing for binding to Endothelial Nitric Oxide Synthase (eNOS) with L-arginine and synthesizing high levels of NO and alteration in NO has also been related to mitochondrial dysfunction, a potential pathogenic mechanism for metabolic dysfunction^38^. Several potential mechanisms may account for the insignificant relationship after follow-up. Firstly, compared with eCO, FeNO may exhibit less involvement in metabolic pathways^30^, since eCO can stimulate insulin and glucagon release from islet cells as well as modulate insulin sensitivity and glucose tolerance in metabolic disorder animals^29^. Secondly, the average level of FeNO in the whole samples is relatively lower when compared with the recommended high risk range: 50 ppb of adults in some studies^1^,^35^, and we also observed a sharp increase of RR for MetS during follow-up when FeNOs were more than 100 ppb in the regression splines. Furthermore, Salonen I. found FeNO were inversely associated with plasma concentration of triglycerides and blood concentration of glycated haemoglobin A1c which was conflicted with this study^39^. The conflicted results might be explained by the difference in study sample: the study sample of Salonen, I’s study was very small and were all ischaemic heart disease patients while our study sample were healthy community residents. And studies indicated that heart diseases might lead to abnormal production of NO and pills to cure heart diseases may leads to the release large quantity of NO^40^.

Although our study examined the relationship between exhaled CO, FeNO and MetS in a cohort study, there are still some limitations. At first, exhaled CO and NO measurements in this study were obtained at a single point, but the use of a standardized protocol and routinely calibrated instrumentation likely served to reduce excess intraindividual and interindividual variability. Secondly, although we adjusted lifestyle factors, like smoking, we could not exclude the effect of ambient air and environment while the concentrations of eCO and FeNO might be influenced by air pollution, home heating apparatus, distance from major roadway, or transportation method, and we also could not accurately calculate current number of cigarettes per day or time between the last cigarette and the CO measurement. However, all the participants lived in two communities from Wuhan city where the ambient air and living conditions were similar, so the effect of environmental factors might be homogenous in the whole population. Thirdly, although diet frequencies for seven kind of food were adjusted in multi-variate models, we cannot evaluate the effect of high-carbonhydrate diet and high-fat diet accurately without accurate information on food intake for every participant. Fourthly, MetS was only assessed at baseline and at 3 years of follow-up and that 40% of individuals in the study sample were not included in the prospective analyses. In addition, all participants in this study are of Chinese Han ethnicity, which minimizes the confounding effects by ethnic background but doesn’t allow us to explore whether the associations of exhaled CO and NO and MetS is different between ethnicities.

## Conclusion

Our study demonstrated that eCO and FeNO were independently and positively associated with the prevalence of MetS, while only eCO was positively and significantly related with the incidence of MetS in nonsmokers prospectively. It may contribute to our new understanding of the importance eCO and FeNO, in the pathogenesis of MetS. Further studies are warranted to confirm our findings and elucidate the potential mechanisms.

## Methods

### Study Sample

In the present study, we used data form a community-based prospective cohort: the Wuhan-Zhuhai (WHZH) cohort study (established in 2011, n = 4812). The design and enrollment criteria of this cohort have been well described previously^41^. In this cohort, standard questionnaires, anthropometry and physical examination were administered at baseline and three years later to collect and update information on lifestyles and occurrence of diseases. For the cross-sectional investigation, we excluded 298 participants with former cardiovascular diseases and lung diseases, as well as 865 people with missing information or outliers (>mean ± 3SD) on indexes of blood test (n = 100), eCO (n = 209) and FeNO (n = 556). After exclusion, a total of 3649 participants were included in our cross-sectional analysis. And, for the longitudinal investigation, we further excluded people lost to follow-up (n = 390), participants with metabolic syndrome (n = 484) at baseline, and individuals with missing information on indexes of blood test (n = 594) at follow-up. Finally, 2,181 participants were included in the longitudinal analysis. However, individuals included and excluded in these analysis were similar with respect to age, sex, body mass index (BMI), and smoking status (*P* > 0.05).

### Ethics Statement

The study protocol was approved by the institutional review boards of Tongji Medical College Institutional review Board, School of Public Health, Tongji Medical College, Huazhong University of Science & Technology (Wuhan, Hubei, China). The methods were carried out in accordance with the approved guidelines. All participants provided written informed consent.

### Exhaled CO and FeNO Assessment

Exhaled CO and FeNO were measured following the standard protocol in resting state in every participant. Exhaled CO was measured for each participant with a MicroCO Meter (Carefusion, Kent, UK). After a 5-minute rest, the participants were instructed to inspire fully and hold the breath for 20 seconds, then seal their lips around the mouthpiece and exhale slowly and fully. The calibrations were performed with standard gas weekly. And FeNO was tested by a Nano Coulomb Nitric Oxide Analyzer (SV-02E, Sunvou Medical Electronics CO., Ltd., Wuxi, China) following the recommendations of American Thoracic Society/European Respiratory Society (2005)^1^. The tests were conducted at least 2 hours after food eating and 30 minutes after strenuous exercise. Calibrations were performed with standard bottle gas (15, 75 and 150 ppb) on a weekly basis.

### Ascertainment of Metabolic Syndrome

MetS was defined according to the diagnostic criteria proposed by the Adult Treatment Program III of the National Cholesterol Education Program (NCEP ATP III, 2005). Participants were defined as MetS patients if they met three or more of the following variables and cutoff points: (1) Fasting triglyceride ≥1.69 mmol/L (150 mg/dL); (2) HDL cholesterol: Men <1.04 mmol/L (40 mg/dL), Women <1.29 mmol/L (50 mg/dL); (3) Fasting glucose: ≥5.5 mmol/L (100 mg/dL); (4) Waist circumference: men ≥102 cm, women ≥88 cm; (5) Systolic blood pressure ≥130 mmHg and/or diastolic blood pressure ≥85 mmHg. And MetS was only ascertained at baseline and at 3 years of follow-up.

### Ascertainment of Covariates

Physical activities contain many aspects of activities, such as, climbing, walking, dancing, cycling, running, swimming and so on. Physical activity is defined as “yes” if the participant exercises ≥2 times per week and each time ≥20 minutes. Diet frequency include the consumption frequency of seven main kind of food: grain, coarse, fruits and vegetables, meat and poultry, fishery product, egg and milk, bacon. The frequency was recorded in 4 categories: times per day, times per week, times per month and times per year in questionnaires and then we transformed consumption frequency of these seven kind of food into times per month in the final analysis respectively.

### Statistical Analyses

Distribution of information on demographic, lifestyles, and some biochemical markers were demonstrated according to quartile of eCO and FeNO, separately. The quartiles of eCO and FeNO were determined using whole number approximate quartiles based on log-transformed values. And the *P*-Value for trend was tested by linear regression for continuous variables and Chi-square tests for category variables. eCO and FeNO were log-transformed in all analysis because of abnormal distribution.

Logistic regression model was used to calculate the odds ratio (OR), risk ratio (RR) and 95% confidence interval (CI) for MetS according to quartile of eCO and FeNO with the lowest quartile as the referent. In model 1, we adjusted for sex and age (continuous). In model 2, we adjusted for age (continuous), sex, body mass index (BMI) (continuous), race (Han, others), marital status (single or divorced, married), education (junior high school or below, senior high school or above), current drinking status (no, yes), diet frequency (times per month, for the following seven kind of food: grain, coarse, fruits and vegetables, meat and poultry, fishery product, egg and milk, bacon ) and physical activity (no, yes). In model 3, we further adjusted for passive smoking (no, yes) and current smoking status (no, yes) as well as variables in model 2. All *p*-values were two sided with a significant level at 0.05, and data were analyzed with SAS 9.1 (SAS Institute Inc. Cary, NC, USA). Rug plots displaying the distribution of eCO and FeNO were generated with R software (R 3.2.1). The display of multivariable-adjusted RR of MetS versus eCO and FeNO was generated with SAS 9.1 using spline regression, the knots were placed at the 5%, 25%, 50%, 75% and 95%^42^.

## Additional Information

**How to cite this article**: Guo, Y. *et al.* Associations of Exhaled Carbon Monoxide and Fractional Exhaled Nitric Oxide with Metabolic Syndrome: A cohort study. *Sci. Rep.* **6**, 24532; doi: 10.1038/srep24532 (2016).

## Supplementary Material

Supplementary Information

## Figures and Tables

**Table 1:** Baseline Characteristics of Participants according to quartiles of eCO and FeNO (Cross-Sectional).

Variables	Total (*n* = 3649)	eCO (ppm)				FeNO (ppb)			
		Q1(*n* = 1159)	Q2(*n* = 816)	Q3(*n* = 808)	Q4(*n* = 866)	Q1(*n* = 822)	Q2(*n* = 958)	Q3(*n* = 930)	Q4(*n* = 939)
**Median(range)**		1(0,2)	3(3,4)	6(5,9)	18(10,98)	9.82(0.24,13.54)	17.25(13.55,21.02)	25.13(21.04,30.75)	39.47(30.76,130.46)
**Age (Mean **±** SD)**	52.70 ± 13.35	52.15 ± 13.75	53.36 ± 13.16	53.63 ± 13.73	51.94 ± 12.56	51.08 ± 12.66	50.81 ± 13.26	53.38 ± 13.40	55.52 ± 13.56
**Sex, male, n (%)**	1272 (34.86)	222 (19.15)	199 (24.39)	285 (35.27)	566 (65.36)	182 (22.14)	307 (32.05)	347 (37.31)	436 (46.43)
**BMI (Mean **±** SD)**	23.95 ± 3.43	23.82 ± 3.39	23.89 ± 3.28	23.99 ± 3.37	24.12 ± 3.67	23.75 ± 3.41	23.96 ± 3.55	23.97 ± 3.32	24.11 ± 3.42
**Waist Circumferences (cm)**	81.98 ± 9.90	80.87 ± 9.91	81.46 ± 9.67	82.13 ± 9.66	83.83 ± 10.83	80.74 ± 9.69	81.76 ± 10.02	82.31 ± 9.92	83.13 ± 9.84
**Current Smoking**	667 (18.28)	27 (2.33)	23 (2.82)	100 (12.38)	517 (59.70)	144 (17.52)	203 (21.19)	169 (18.17)	151 (16.08)
**Current Drinking**	533 (14.61)	98 (8.46)	75 (9.19)	109 (13.49)	251 (28.98)	75 (9.12)	127 (13.26)	165 (17.74)	166 (17.68)
**Physical Activity**	1120 (30.69)	353 (30.46)	274 (33.58)	255 (31.56)	238 (27.48)	289 (35.16)	234 (24.43)	285 (30.65)	312 (33.23)
**Blood Pressure (mm Hg)**									
SBP	131.09 ± 20.32	130.84 ± 19.75	130.95 ± 20.29	131.29 ± 20.88	131.24 ± 20.40	130.50 ± 20.57	129.21 ± 19.88	130.73 ± 20.63	133.93 ± 19.96
DBP	77.24 ± 11.53	77.55 ± 11.41	77.21 ± 11.49	76.48 ± 11.37	77.55 ± 11.84	77.11 ± 11.42	76.67 ± 11.65	77.09 ± 11.84	78.09 ± 11.16
**Serum Cholesterol**									
Total Cholesterol (mmol/L)	5.11 ± 1.29	5.06 ± 1.11	5.10 ± 1.61	5.11 ± 1.07	5.19 ± 1.17	5.12 ± 1.13	5.01 ± 1.07	5.08 ± 1.10	5.24 ± 1.74
HDL Cholesterol (mmol/L)	1.55 ± 0.43	1.57 ± 0.44	1.55 ± 0.42	1.56 ± 0.42	1.51 ± 0.45	1.60 ± 0.44	1.51 ± 0.41	1.56 ± 0.45	1.52 ± 0.42
LDL Cholesterol (mmol/L)	3.04 ± 1.03	2.98 ± 0.98	2.96 ± 1.03	3.12 ± 1.07	3.12 ± 1.02	2.86 ± 1.05	3.04 ± 1.00	3.10 ± 0.99	3.16 ± 1.02
**Serum Triglycerides (median, IQR)(mmol/L)**	1.18 (0.81,1.84)	1.12 (0.78,1.65)	1.18 (0.81,1.74)	1.26 (0.81,1.97)	1.27 (0.86,2.02)	1.13 (0.78, 1.66)	1.21 (0.82, 1.91)	1.17 (0.80, 1.79)	1.25 (0.87, 1.93)
**Blood Glucose(mmol/L)**	**4.87 **±** 1.62**	4.79 ± 1.40	4.82 ± 1.64	4.89 ± 1.69	4.99 ± 1.78	4.58 ± 1.81	4.82 ± 1.46	4.94 ± 1.43	5.12 ± 1.69

Abbreviation: eCO, exhaled carbon monoxide; FeNO, fractional exhaled nitric oxide; SD, standard deviation; BMI, body mass index; SBP, systolic blood pressure; DBP, diastolic blood pressure; HDL, high-density lipoprotein; LDL, low-density lipoprotein; IQR: interquartile range.

Values are presented as Mean ± SD or frequency unless otherwise indicated.

**Table 2:** Cross-Sectional Odds Ratios for MetS according to quartiles of eCO and FeNO.

	First Quartile	Second Quartile	Third Quartile	Fourth Quartile	Per 1 log-unit increment
eCO					
Model 1 OR(95%CI)	1.00 (referent)	0.96 (0.79–1.18)	1.00 (0.80–1.22)	1.81 (1.44–2.27)	1.25 (1.15–1.37)
Model 2 OR(95%CI)	1.00 (referent)	1.01 (0.81–1.24)	1.04 (0.85–1.29)	1.82 (1.44–2.28)	1.27 (1.16–1.39)
Model 3 OR(95%CI)	1.00 (referent)	0.99 (0.80–1.23)	1.03 (0.83–1.28)	1.71 (1.34–2.18)	1.22 (1.11–1.35)
FeNO					
Model 1 OR(95%CI)	1.00 (referent)	1.12 (0.90–1.39)	1.01 (0.81–1.26)	1.10 (0.88–1.36)	1.01 (0.89–1.13)
Model 2 OR(95%CI)	1.00 (referent)	1.39 (1.11–1.76)	1.19 (0.94–1.50)	1.42 (1.12–1.80)	1.15 (1.01–1.30)
Model 3 OR(95%CI)	1.00 (referent)	1.37 (1.07–1.74)	1.16 (0.91–1.49)	1.45 (1.13–1.86)	1.14 (1.00–1.30)

Abbreviation: eCO, exhaled carbon monoxide; FeNO, fractional exhaled nitric oxide;

Model 1: adjusted for age (continuous) and sex;

Model 2: adjusted for all variables in Model 1 and body mass index (BMI) (continuous), race (Han, others), marital status (single or divorced, married), education (junior high school or below, senior high school or above), current drinking status (no, yes), diet frequency (times per month, for seven kind of food: grain, coarse, fruits and vegetables, meat and poultry, fishery product, egg and milk, bacon ) and physical activity (no, yes);

Model 3: adjusted for all variables Model 2 and passive smoking (no, yes) and current smoking status (no, yes).

**Table 3:** Risk Ratios for MetS according to quartiles of eCO and FeNO.

	First Quartile	Second Quartile	Third Quartile	Fourth Quartile	Per 1 log-unit increment
eCO					
Model 1 RR(95%CI)	1.00 (referent)	1.29(0.92–1.81)	1.41(1.02–1.94)	1.47(1.06–2.03)	1.16(1.00–1.35)
Model 2 RR(95%CI)	1.00 (referent)	1.28(0.89–1.85)	1.42(1.01–2.01)	1.44(1.01–2.03)	1.15(1.00–1.36)
Model 3 RR(95%CI)	1.00 (referent)	1.23(0.87–1.74)	1.35(1.01–1.82)	1.48(1.06–2.06)	1.12(1.02–1.26)
FeNO					
Model 1 RR(95%CI)	1.00 (referent)	1.35(0.97–1.89)	1.13(0.80–1.60)	1.19(0.84–1.68)	1.20(0.95–1.51)
Model 2 RR(95%CI)	1.00 (referent)	1.37(0.96–1.96)	1.26(0.88–1.82)	1.18(0.81–1.71)	1.25(0.98–1.59)
Model 3 RR(95%CI)	1.00 (referent)	1.41(0.98–2.03)	1.30(0.90–1.89)	1.22(0.83–1.79)	1.23(0.97–1.57)

Abbreviation: eCO, exhaled carbon monoxide; FeNO, fractional exhaled nitric oxide;

Model 1: adjusted for age (continuous) and sex;

Model 2: adjusted for all variables in Model 1 and body mass index (BMI) (continuous), race (Han, others), marital status (single or divorced, married), education (junior high school or below, senior high school or above), current drinking status (no, yes), diet frequency (times per month, for seven kind of food: grain, coarse, fruits and vegetables, meat and poultry, fishery product, egg and milk, bacon ) and physical activity (no, yes);

Model 3: adjusted for all variables Model 2 and passive smoking (no, yes), current smoking status (no, yes).
