# Supplementary material for: Associations of Exhaled Carbon Monoxide and Fractional Exhaled Nitric Oxide with Metabolic Syndrome: A Cohort Study
Source: Sci Rep. 2016 Apr 14;6:24532. doi: 10.1038/srep24532 (PMC4830973; doi:10.1038/srep24532)

Associations of Exhaled Carbon Monoxide and Fractional Exhaled Nitric Oxide with Metabolic Syndrome：A cohort study

Yanjun Guo, Jixuan Ma, Wei Lu, Jintong He, Runbo Zhang, Jing Yuan, Weihong Chen

SUPPLEMENTARY MATERIALS

Supplementary Table S1 Characteristics of Participants Included and Excluded of Longitudinal Analysis

Supplementary Table S2 Adjusted ORs for cross-sectional eCO and FeNO levels associated with MetS stratified by gender and smoking status

Supplementary Table S3 Adjusted RRs for eCO and FeNO levels associated with MetS stratified by gender and smoking status.

Supplementary Figure S1 A rug plot displaying the frequency distribution of exhaled CO in the study sample.

Supplementary Figure S2 A rug plot displaying the frequency distribution of exhaled NO in the study sample.

Supplementary Figure S3 ROC Curves for eCO and FeNO at Follow-up.

Supplementary Figure S4 Multivariable-adjusted spline graph displaying the relation of eCO (A) and FeNO (B) with MetS cross-sectionally.

Supplementary Table S1 Characteristics of Participants Included and Excluded of Longitudinal Analysis

Variables	Included	Excluded	*P* Value
**Age (Mean ±SD)**	52.98 ±11.87	52.48 ±14.11	0.08
**Sex, male, n (%)**	766(35.12)	506(34.47)	0.53
**BMI (Mean ±SD)**	23.61 ±3.08	24.14 ±3.60	0.07
**Waist Circumferences (cm)**	82.05 ±10.31	81.49 ±9.07	0.00
**Current Smoking**	398(18.28)	268(18.27)	0.77
**Current Drinking**	336(15.41)	197(13.48)	0.08
**Physical Activity**	681(31.22)	439(29.89)	0.31
**Blood Pressure (mm Hg)**			
SBP	130.1 ±18.89	131.2 ±21.03	0.04
DBP	76.45 ±10.82	77.68 ±11.89	0.00
**Serum Cholesterol**			
Total Cholesterol (mmol/L)	5.14 ±1.51	5.10 ±1.15	0.32
HDL Cholesterol (mmol/L)	1.61 ±0.43	1.51 ±0.43	0.00
LDL Cholesterol (mmol/L)	3.09 ±0.99	3.01 ±1.04	0.02
**Serum Triglycerides (median,IQR)(mmol/L)**	1.09(0.79-1.49)	1.28(0.83-2.00)	0.00
**Blood Glucose(mmol/L)**	4.72 ±1.12	4.98 ±1.83	0.00

Abbreviation: SD, standard deviation; BMI, body mass index; SBP, systolic blood pressure; DBP, diastolic blood pressure; HDL, high-density lipoprotein; LDL, low-density lipoprotein; IQR: interquartile range.

Values are presented as Mean ±SD or frequency unless otherwise indicated.

P Value of continuous variables was calculated with linear regression without adjustments; *P* Value of category variables was calculated with Chi-square tests.

Supplementary Table S2 Adjusted ORs for cross-sectional eCO and FeNO levels associated with MetS stratified by gender and smoking status

	First Quartile	Second Quartile	Third Quartile	Fourth Quartile	*P*-trend
**Gender**					
**Male**					
**eCO RR(95%CI)**	1.00 (referent)	0.90(0.38-2.13)	1.48(0.70-2.61)	1.34(1.08-2.23)	0.26
**FeNO RR(95%CI)**	1.00 (referent)	1.80(0.76-4.26)	1.29(0.54-3.08)	2.06(1.02-3.62)	0.13
**Female**					
**eCO RR(95%CI)**	1.00 (referent)	1.19(0.92-1.53)	1.35(1.04-1.76)	1.17(1.05-1.41)	0.08
**FeNO RR(95%CI)**	1.00 (referent)	1.59(1.21-2.09)	1.37(1.04-1.81)	1.51(1.13-2.01)	0.01
**Smoke**					
**Smokers**					
**eCO RR(95%CI)**	1.00 (referent)	/	0.94(0.17-5.02)	1.27(0.28-5.82)	0.24
**FeNO RR(95%CI)**	1.00 (referent)	1.99(0.85-4.68)	1.21(0.47-3.10)	2.21(1.00-5.43)	0.19
**Non-smokers**					
**eCO RR(95%CI)**	1.00 (referent)	1.18(0.93-1.49)	1.41(1.11-1.80)	1.21(1.04-1.65)	0.06

**FeNO**	**RR(95%CI)**	1.00 (referent)	1.61(1.24-2.10)	1.40(1.08-1.83)	1.59(1.21-2.08)	0.01

Abbreviation: eCO, exhaled carbon monoxide; FeNO, fractional exhaled nitric oxide;

Adjusted for age (continuuso), gender, body mass index (BMI) (continuous), race (Han, others), marital status (single or divorced, married), education (junior high school or below, senior high school or above), current drinking status (no, yes), diet frequency (times per month, for seven kind of food: grain, coarse, fruits and vegetables, meat and poultry, fishery product, egg and milk, bacon) and physical activity (no, yes), passive smoking (no, yes), and current smoking status (no, yes).

Supplementary Table S3 Adjusted RRs for eCO and FeNO levels associated with MetS stratified by gender and smoking status

		First Quartile	Second Quartile	Third Quartile	Fourth Quartile	*P*-trend
**Gender**						
**Male**						
**eCO**	**RR(95%CI)**	1.00 (referent)	1.22(0.60--2.50)	1.84(0.93--3.62)	1.93(1.02-3.60)	0.01
**FeNO**	**RR(95%CI)**	1.00 (referent)	1.19(0.63--2.27)	1.20(0.63--2.33)	1.28(0.67--2.44)	0.47
**Female**						
**eCO**	**RR(95%CI)**	1.00 (referent)	1.01(0.66--1.53)	1.26(0.78--2.05)	1.57(1.03--2.37)	0.02
**FeNO**	**RR(95%CI)**	1.00 (referent)	1.79(1.15--2.86)	1.51(0.96--2.38)	1.30(0.82--2.08)	0.43
**Smoke**						
**Smokers**						
**eCO**	**RR(95%CI)**	1.00 (referent)	1.10(0.42--2.90)	1.18(0.47--2.98)	1.53(0.61--3.83)	0.36
**FeNO**	**RR(95%CI)**	1.00 (referent)	1.20(0.44--2.75)	1.17(0.48--2.85)	1.26(0.54--2.84)	0.25

Non-smokers

**eCO**	**RR(95%CI)**	1.00 (referent)	1.16(0.80--1.69)	1.26(0.83--1.93)	1.36(1.04--1.85)	0.01
**FeNO**	**RR(95%CI)**	1.00 (referent)	1.62(1.11--2.35)	1.26(0.86--1.86)	1.38(0.94--2.02)	0.10

Abbreviation: eCO, exhaled carbon monoxide; FeNO, fractional exhaled nitric oxide;

Adjusted for age (continuuso), gender, body mass index (BMI) (continuous), race (Han, others), marital status (single or divorced, married), education (junior high school or below, senior high school or above), current drinking status (no, yes), diet frequency (times per month, for seven kind of food: grain, coarse, fruits and vegetables, meat and poultry, fishery product, egg and milk, bacon) and physical activity (no, yes), passive smoking (no, yes), and current smoking status (no, yes).

Supplementary Figure S1 A rug plot displaying the frequency distribution of exhaled CO in the study sample.

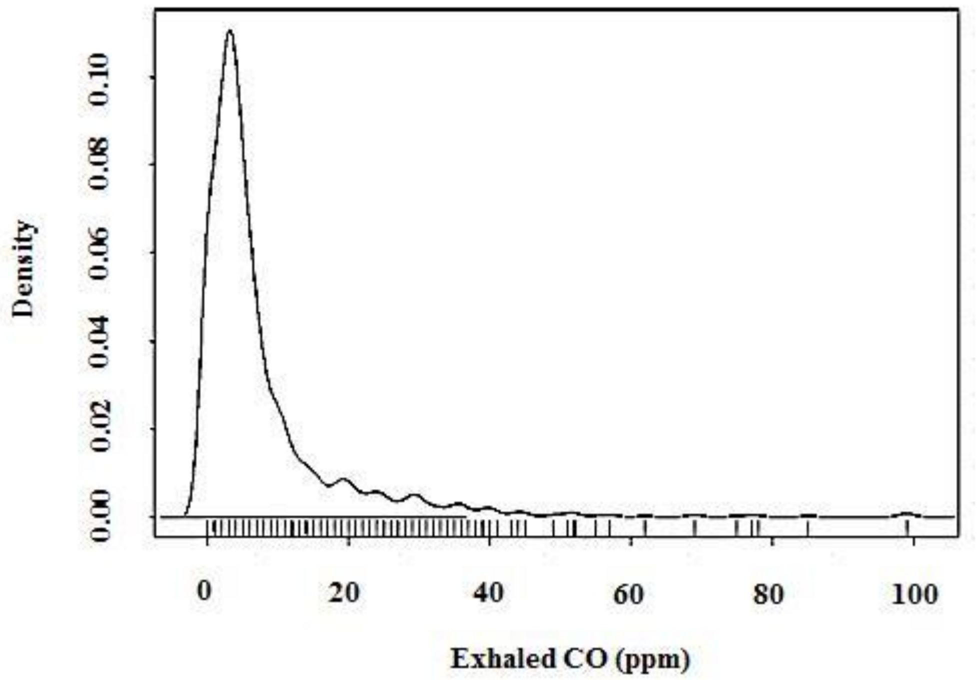

Supplementary Figure S2 A rug plot displaying the frequency distribution of exhaled NO in the study sample.

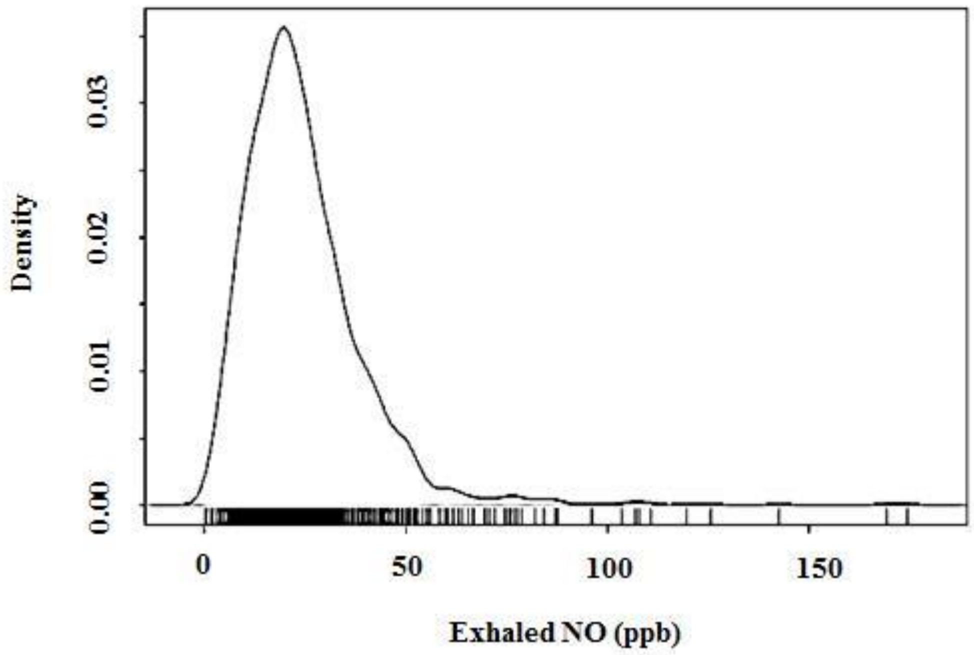

Supplementary Figure S3 ROC Curves for eCO and FeNO at Follow-up.

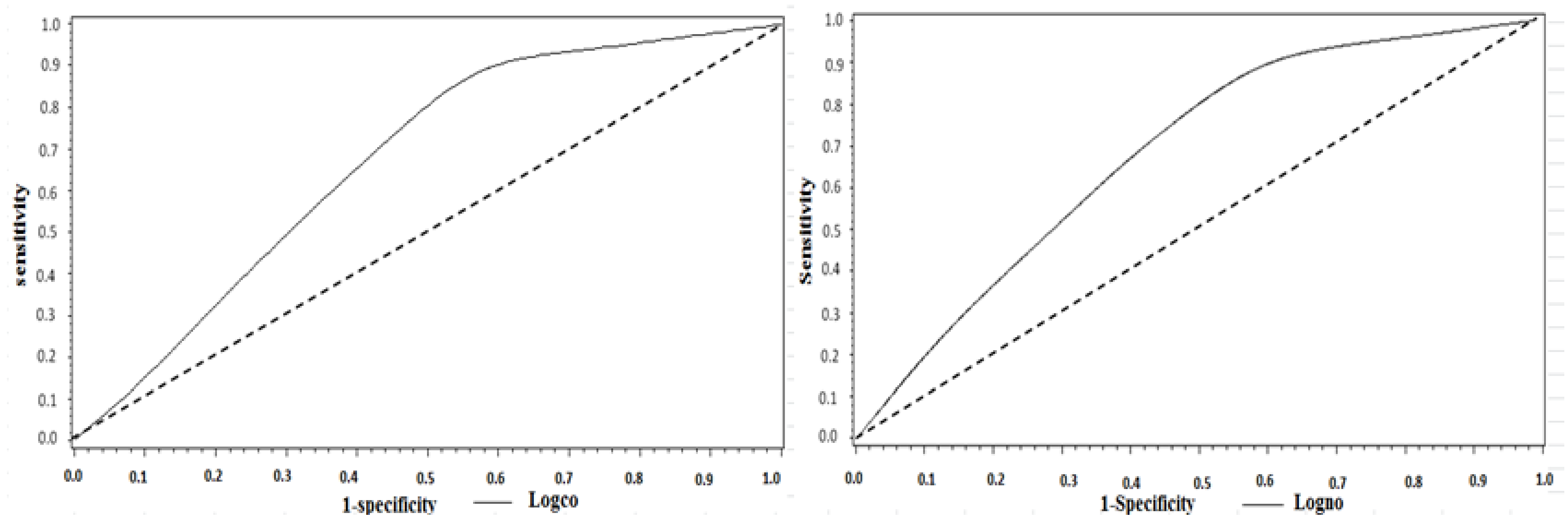

Supplementary Figure S4 Multivariable-adjusted spline graph displaying the relation of eCO (A) and FeNO (B) with MetS cross-sectionally.

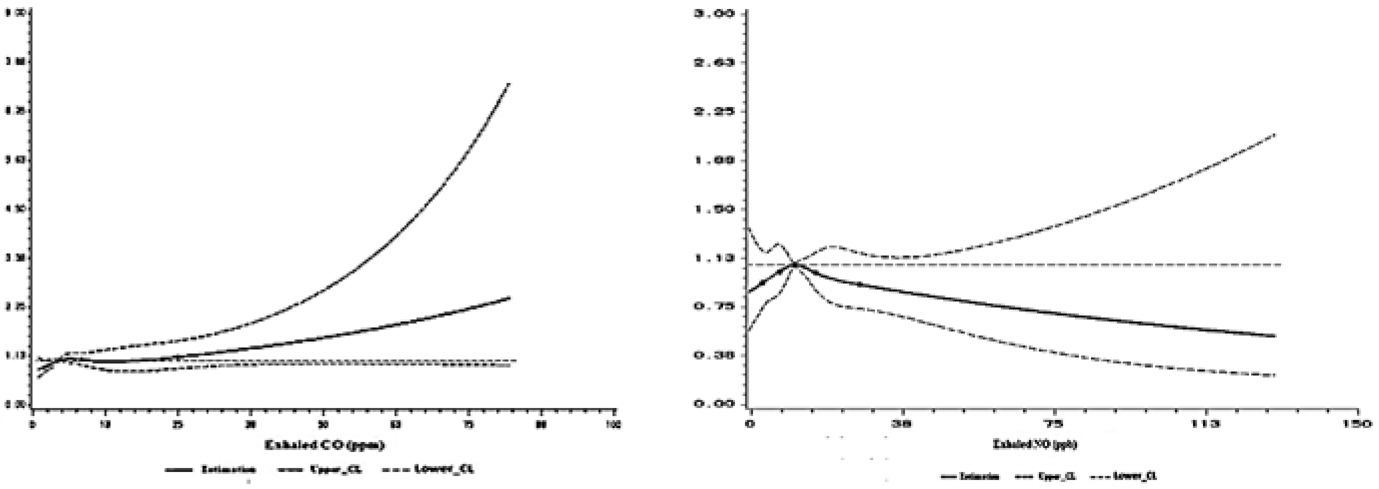

Supplement: Supplementary Information [file srep24532-s1.pdf]
